# Predictors of social risk for post-ischemic stroke reintegration

**DOI:** 10.1038/s41598-024-60507-7

**Published:** 2024-05-02

**Authors:** Katryna K. Cisek, Thi Nguyet Que Nguyen, Alejandro Garcia-Rudolph, Joan Saurí, Helard Becerra Martinez, Andrew Hines, John D. Kelleher

**Affiliations:** 1 AIDHM, Artificial Intelligence in Digital Health and Medicine, Technological University Dublin, Dublin, Ireland; 2 Universitat Autònoma de Barcelona, Cerdanyola del Vallès, Bellaterra, Spain; 3 Fundació Institute d’Investigació en Ciències de la Salut Germans Trias i Pujol, Badalona, Spain; 4 Institut Guttmann Hospital de Neurorehabilitacio, Badalona, Spain; 5 STRATIF-AI, Continuous stratification for improved prevention, treatment, and rehabilitation of stroke patients using digital twins and AI, Horizon Europe Project, Linköping, Sweden; 6 School of Computer Science, University College Dublin, Dublin, Ireland; 7 ADAPT Research Centre, School of Computer Science and Statistics, Trinity College Dublin, Dublin, Ireland; 8 RESQ+, Comprehensive solutions of healthcare improvement based on the global Registry of Stroke Care Quality, Horizon Europe Project, Brno, Czech Republic

**Keywords:** Stroke, Rehabilitation, Reintegration, Machine learning, Social risk, Prediction model, SHAP analysis, Socioeconomic support, Computational biology and bioinformatics, Neurology

## Abstract

After stroke rehabilitation, patients need to reintegrate back into their daily life, workplace and society. Reintegration involves complex processes depending on age, sex, stroke severity, cognitive, physical, as well as socioeconomic factors that impact long-term outcomes post-stroke. Moreover, post-stroke quality of life can be impacted by social risks of inadequate family, social, economic, housing and other supports needed by the patients. Social risks and barriers to successful reintegration are poorly understood yet critical for informing clinical or social interventions. Therefore, the aim of this work is to predict social risk at rehabilitation discharge using sociodemographic and clinical variables at rehabilitation admission and identify factors that contribute to this risk. A Gradient Boosting modelling methodology based on decision trees was applied to a Catalan 217-patient cohort of mostly young (mean age 52.7), male (66.4%), ischemic stroke survivors. The modelling task was to predict an individual’s social risk upon discharge from rehabilitation based on 16 different demographic, diagnostic and social risk variables (family support, social support, economic status, cohabitation and home accessibility at admission). To correct for imbalance in patient sample numbers with high and low-risk levels (prediction target), five different datasets were prepared by varying the data subsampling methodology. For each of the five datasets a prediction model was trained and the analysis involves a comparison across these models. The training and validation results indicated that the models corrected for prediction target imbalance have similarly good performance (AUC 0.831–0.843) and validation (AUC 0.881 - 0.909). Furthermore, predictor variable importance ranked social support and economic status as the most important variables with the greatest contribution to social risk prediction, however, sex and age had a lesser, but still important, contribution. Due to the complex and multifactorial nature of social risk, factors in combination, including social support and economic status, drive social risk for individuals.

## Introduction

Following post-stroke rehabilitation, the long-term patient outcome generally encompasses reintegration into normal activities of daily living in the home, community, and workplace^1–3^. An essential part of this process is community integration, which includes relationships with others, the ability to be independent in daily life activities (ADL), and participation in meaningful events^4–6^. There is consistent evidence that continued positive interaction with one’s proximate social environment (e.g., family, friends and work life) exerts beneficial effects on health and well-being, increasing resilience to unexpected setbacks^7,8^. Conversely, social isolation or lack of close social ties is associated with poor health and increased mortality risk^9,10^. Complementary to community integration is minimizing social risk, which is a complex and multifactorial phenomenon that can vary significantly for an individual, but generally encompasses environmental, socioeconomic, as well as family and social support factors^11–13^. For example, a patient with insufficient family support who is unable to access social support, such as home health care or a day center, is at a greater risk of poorer quality of life during reintegration, social isolation, and retreat from life (also termed fragility). These considerations emphasize the importance of quality of life, social well-being, as well as adequate support for patients with social risks during long-term reintegration^14,15^.

Several studies in the literature highlight the importance of family support in the context of the social environment (also termed sociofamiliar) and the socioeconomic situation in the overall rehabilitation outcome and reintegration of patients^16–19^. Although these studies target a broader patient population with physical, cognitive and sensory disturbances which include stroke patients, as well as elderly patients and their likelihood of discharge from a geriatric unit centre, nevertheless sociofamiliar factors play a significant role in the resilience of most patient populations. Ramírez-Duque et al. analyzed the clinical, functional, cognitive, sociofamiliar, and other characteristics of pluripathological patients and found that older people with cognitive and more severe functional impairment had worse sociofamiliar support than other patient groups^18^. In a similarly comprehensive study of the clinical, functional and social risk profiles of the elderly in a community in Lima, Peru, Varela-Pinedo et al. found that 8% of individuals lived alone, and nearly 60% had inadequate socioeconomic support and were at social risk^19^. In another study, Cahuana-Cuentas Milagros et al. concluded that family and socioeconomic factors have a significant impact on the levels of resilience of people with physical and sensory disabilities^17^. Finally, Sabartés et al. identified a deteriorated social situation as the only significant predictor of being institutionalized rather than discharged home for a cohort of hospitalized elderly patients^16^.

Since family, social and economic factors have been identified as having a significant impact on the quality of life of patients post-rehabilitation, the key goals of post-stroke reintegration have focused on improving patient outcomes across these factors, as well as designing personalized interventions for patients with social risk^20^. More recently, special situations, such as the pandemic, have added additional uncertainties and strains to the recovery and reintegration process of patients^21–23^. Therefore, it is essential for both the patients as well as clinicians to be able to forecast the level of dependence on social supports (the level of social risk) for an individual patient at admission to rehabilitation so that the necessary interventions can be put in place during rehabilitation in order to prevent setbacks after discharge. Due to the complexity of reintegration, encompassing the spatiotemporal component (long-term processes taking place in the home, community, and workplace)^24^, multifactorial component (interdependency of psychosocial, environmental, and socioeconomic factors) as well as demographic and cultural factors (younger age, gender, geographic location)^25–29^, predictive modelling of social risk is an invaluable tool in not only forecasting the level of social risk for an individual but also identifying the contributing factors to this risk. Accurate predictions of factors contributing to social risk can allow rehabilitation professionals (social workers, physical therapists, neuropsychogists, psychologists, etc.) to support persons with personalized interventions, prevent fragility, as well as help improve patients’ quality of life and support their specific clinical needs and challenges throughout the reintegration process. For this purpose, machine learning (ML) algorithms and statistical analyses have been employed in recent years to develop predictive models for stroke reintegration, such as in the case of long-term trajectories of community integration^30,31^, and functional and cognitive improvement during rehabilitation^32,33^. However, predictive modelling for social risk utilizing ML methodology has been a largely underexplored topic^34^. Cisek, et al. focused on various conceptualizations of social risk during post-stroke reintegration, such as the International Classification of Functioning, Disability, and Health (ICF) framework, as well as utilizing data visualization to explore the cohort^35^. In this work, we go beyond data exploration and understanding to predictive modeling and apply machine learning to develop interpretable predictive models that provide individualized predictions to guide personalized interventions for patients with social risk.

**Table 1:** EVSF questionnaire items and risk scoring metric.

Items	Level of risk	Scores
Cohabitation	Lives with family/core of coexistence or stable partner	1
	Lives in a residence in stable situation	2
	Lives alone, but with a close family circle (children, siblings)	3
	Lives with non-relatives or with person with disability or chronic disease	4
	Lives alone, no relatives close	5
Economic status	With sufficient and stable incomes	1
	With stable but insufficient incomes	2
	With minimum incomes (non-contributory benefit)	3
	With fix incomes received in non-regular basis	4
	With no fix incomes received	5
Home access	Appropriate to your needs	1
	Architectural barriers with possibilities for adaptation	2
	Architectural barriers without possibility of adaptation	3
	Cannot return home	4
	No home	5
Family support	Autonomous/no support needed from family/core of coexistence	1
	Family/core of coexistence is able to provide the required support	2
	Family/core of coexistence & limited capacity of providing support	3
	Rejected or abandoned by family or by core of coexistence	4
	No family/ No core of coexistence	5
Social support	Autonomous or with enough informal support	1
	Not enough social support, but can afford private services	2
	Not enough social support, needs proximity public services (e.g., home health care, day centre)	3
	Needs public institutional alternative (e.g., long term sociosanitary centre or assisted residence)	4
	Can’t access public support (e.g., foreigner without residence card)	5

## Methodology

### Social risk questionnaire

Social workers conduct an interview at admission and discharge from the rehabilitation hospital following a structured questionnaire to assess social risk of patients, called “Escala de Valoracion Socio Familiar” (EVSF; *eng. trans.:* sociofamiliar assessment scale)^35^. The questionnaire is based on the Gijon sociofamiliar scale^36^ that includes five items (housing, family situation, economic situation, relationships, and social support). Accordingly, the EVSF questionnaire consists of five items also termed dimensions: cohabitation, economic status (indicating income sufficiency), home status (indicating home accessibility in case of mobility problems), family support and social support (Table 1). Each of these five items has five levels of risk that are scored from 1 to 5. A higher score for each item represents a higher risk for the social reintegration of the patient. The total score is the sum of the five-item scores and is between 5 and 25 and determines four social risk categories: (i) no social risk (5 points); (ii) mild social risk (6-9 points); (iii) important social risk (10-14 points); and (iv) severe social risk (15-25 points)^35^. The reliability and validity of this questionnaire were evaluated by comparing the score obtained on the scale with a reference criterion of an independent, blind assessment by social work experts. It was reported to enable the detection of risk situations and social problems with good reliability and acceptable validity^37^.

**Table 2:** Training set patient cohort admission data including social risk and demographics.

	GREEN (N = 155)	RED (N = 62)	Overall (N = 217)
Sex			
Male	101 (65.2%)	43 (69.4%)	144 (66.4%)
Female	54 (34.8%)	19 (30.6%)	73 (33.6%)
Age @ stroke (years)			
Mean (SD)	53.2 (10.8)	51.6 (8.03)	52.7 (10.1)
Median [Min, Max]	53.4 [19.2, 83.8]	53.0 [33.9, 80.9]	53.4 [19.2, 83.8]
Days since stroke			
Mean (SD)	45.7 (24.7)	58.8 (29.0)	49.5 (26.6)
Median [Min, Max]	38.8 [8.74, 124]	49.5 [11.6, 124]	41.8 [8.74, 124]
Stroke type			
Embolic	36 (23.2%)	10 (16.1%)	46 (21.2%)
Others	47 (30.3%)	26 (41.9%)	73 (33.6%)
Thrombolic	72 (46.5%)	26 (41.9%)	98 (45.2%)
Length of stay (days)			
Mean (SD)	94.2 (33.1)	92.5 (29.4)	93.7 (32.0)
Median [Min, Max]	93.0 [35.0, 149]	88.0 [35.0, 139]	90.0 [35.0, 149]
Education level			
High	83 (53.5%)	26 (41.9%)	109 (50.2%)
Low	72 (46.5%)	36 (58.1%)	108 (49.8%)
Civil status			
Married	98 (63.2%)	32 (51.6%)	130 (59.9%)
Not married	57 (36.8%)	30 (48.4%)	87 (40.1%)
NIHSS			
Mean (SD)	13.2 (5.98)	13.7 (6.19)	13.3 (6.03)
Median [Min, Max]	12.0 [1.00, 26.0]	15.0 [1.00, 26.0]	12.0 [1.00, 26.0]
Cognitive FIM			
Mean (SD)	24.7 (8.66)	21.2 (8.90)	23.7 (8.86)
Median [Min, Max]	27.0 [5.00, 35.0]	23.0 [5.00, 35.0]	25.0 [5.00, 35.0]
Motor FIM			
Mean (SD)	47.1 (21.7)	39.0 (20.9)	44.8 (21.8)
Median [Min, Max]	47.0 [13.0, 91.0]	34.0 [13.0, 84.0]	45.0 [13.0, 91.0]
Total FIM			
Mean (SD)	71.8 (27.5)	60.2 (26.8)	68.5 (27.7)
Median [Min, Max]	74.0 [18.0, 122]	57.5 [19.0, 111]	70.0 [18.0, 122]
Cohabitation			
Median [Min, Max]	1.00 [1.00, 5.00]	1.00 [1.00, 5.00]	1.00 [1.00, 5.00]
Economic status			
Median [Min, Max]	1.00 [1.00, 5.00]	2.00 [1.00, 5.00]	1.00 [1.00, 5.00]
Home access			
Median [Min, Max]	2.00 [1.00, 5.00]	2.00 [1.00, 5.00]	2.00 [1.00, 5.00]
Family support			
Median [Min, Max]	2.00 [1.00, 5.00]	3.00 [1.00, 5.00]	2.00 [1.00, 5.00]
Social support			
Median [Min, Max]	1.00 [1.00, 4.00]	3.00 [1.00, 5.00]	2.00 [1.00, 5.00]

Statistics of patients with negligible social risk (GREEN) and significant social risk (RED) including counts and percentages, the Mean (average value), Median (middle value with minimum and maximum value ranges) and Standard deviation (SD). The Civil Status of non-married patients includes single, separated, divorced, and widowed individuals. Education Level low level includes illiteracy, basic ability to read and write and primary schooling, whereas high education indicates secondary schooling, college, and advanced degree.

*FIM* functional independence measure, *NIHSS* National Institutes of Health Stroke Scale.

**Table 3:** Hold-out test set cohort information including social risk and demographics.

	GREEN (N = 84)	RED (N = 33)	Overall (N = 117)
Sex			
Female	21 (25.0%)	10 (30.3%)	31 (26.5%)
Male	63 (75.0%)	23 (69.7%)	86 (73.5%)
Age @ stroke (years)			
Mean (SD)	51.9 (11.3)	51.3 (6.21)	51.8 (10.1)
Median [Min, Max]	50.8 [14.1, 85.8]	52.3 [39.4, 67.1]	51.0 [14.1, 85.8]
Days since stroke			
Mean (SD)	78.2 (96.7)	105 (101)	85.7 (98.2)
Median [Min, Max]	46.5 [14.0, 605]	68.0 [26.0, 419]	58.0 [14.0, 605]
Stroke type			
Embolic	19 (22.6%)	12 (36.4%)	31 (26.5%)
Others	30 (35.7%)	9 (27.3%)	39 (33.3%)
Thrombolic	35 (41.7%)	12 (36.4%)	47 (40.2%)
Length of stay (days)			
Mean (SD)	139 (83.0)	124 (73.1)	135 (80.4)
Median [Min, Max]	153 [11.0, 468]	117 [27.0, 341]	151 [11.0, 468]
Education level			
High	50 (59.5%)	18 (54.5%)	68 (58.1%)
Low	34 (40.5%)	15 (45.5%)	49 (41.9%)
Civil status			
Married	52 (61.9%)	19 (57.6%)	71 (60.7%)
Not married	32 (38.1%)	14 (42.4%)	46 (39.3%)
NIHSS			
Mean (SD)	12.8 (6.17)	15.3 (5.28)	13.5 (6.02)
Median [Min, Max]	13.0 [0, 26.0]	17.0 [5.00, 27.0]	14.0 [0, 27.0]
Cognitive FIM			
Mean (SD)	23.3 (9.32)	20.0 (8.36)	22.4 (9.15)
Median [Min, Max]	25.0 [5.00, 35.0]	22.0 [5.00, 35.0]	23.0 [5.00, 35.0]
Motor FIM			
Mean (SD)	42.8 (24.6)	35.8 (20.7)	40.8 (23.7)
Median [Min, Max]	37.5 [13.0, 89.0]	31.0 [13.0, 78.0]	36.0 [13.0, 89.0]
Total FIM			
Mean (SD)	66.1 (31.1)	55.8 (25.4)	63.2 (29.8)
Median [Min, Max]	62.5 [19.0, 124]	53.0 [18.0, 103]	59.0 [18.0, 124]
Cohabitation			
Median [Min, Max]	1.00 [1.00, 5.00]	1.00 [1.00, 5.00]	1.00 [1.00, 5.00]
Economic status			
Median [Min, Max]	1.00 [1.00, 5.00]	2.00 [1.00, 5.00]	1.00 [1.00, 5.00]
Home access			
Median [Min, Max]	2.00 [1.00, 5.00]	3.00 [1.00, 5.00]	2.00 [1.00, 5.00]
Family support			
Median [Min, Max]	2.00 [1.00, 5.00]	3.00 [2.00, 5.00]	3.00 [1.00, 5.00]
Social support			
Median [Min, Max]	2.00 [1.00, 4.00]	3.00 [1.00, 4.00]	3.00 [1.00, 4.00]

Statistics of patients with negligible social risk (GREEN) and significant social risk (RED) including counts and percentages, the Mean (average value), Median (middle value with minimum and maximum value ranges) and Standard deviation (SD). The Civil Status of non-married patients includes single, separated, divorced, and widowed individuals. Education Level low level includes illiteracy, basic ability to read and write and primary schooling, whereas high education indicates secondary schooling, college, and advanced degree.

*FIM* functional independence measure, *NIHSS* National Institutes of Health Stroke Scale.

### Training set patient cohort

Demographic, diagnostic and questionnaire data utilizing the EVSF items during the rehabilitation and reintegration of patients were recorded and collected at the Institut Guttmann (Barcelona, Spain) from 2007 to 2020. Inclusion criteria for this cohort consisted of adult patients 18–85 years of age at the time of stroke with an ischemic stroke diagnosis who were admitted within 3 weeks of the onset of symptoms, without any previous comorbidities leading to disability, and whose data was recorded within a week of admission and discharge. Exclusion criteria were any of the following: diagnosis of stroke in the context of another concomitant comorbidity (e.g., traumatic brain injury), a previous history of another disabling condition, patients with EVSF questionnaire performed more than 5 months post-injury, as well as more than 5 months stay at the rehabilitation hospital. The authors confirm that this study is compliant with the Helsinki Declaration of 1975, as revised in 2008 and it was approved by the Ethics Committee of Clinical Research of Institut Guttmann. Experimental protocols applied in this study were approved by Institut Guttmann’s Ethics Commitee. At admission participants provided written informed consent to be included in research studies addressed by the Institut Guttmann hospital.

**Figure 1:**
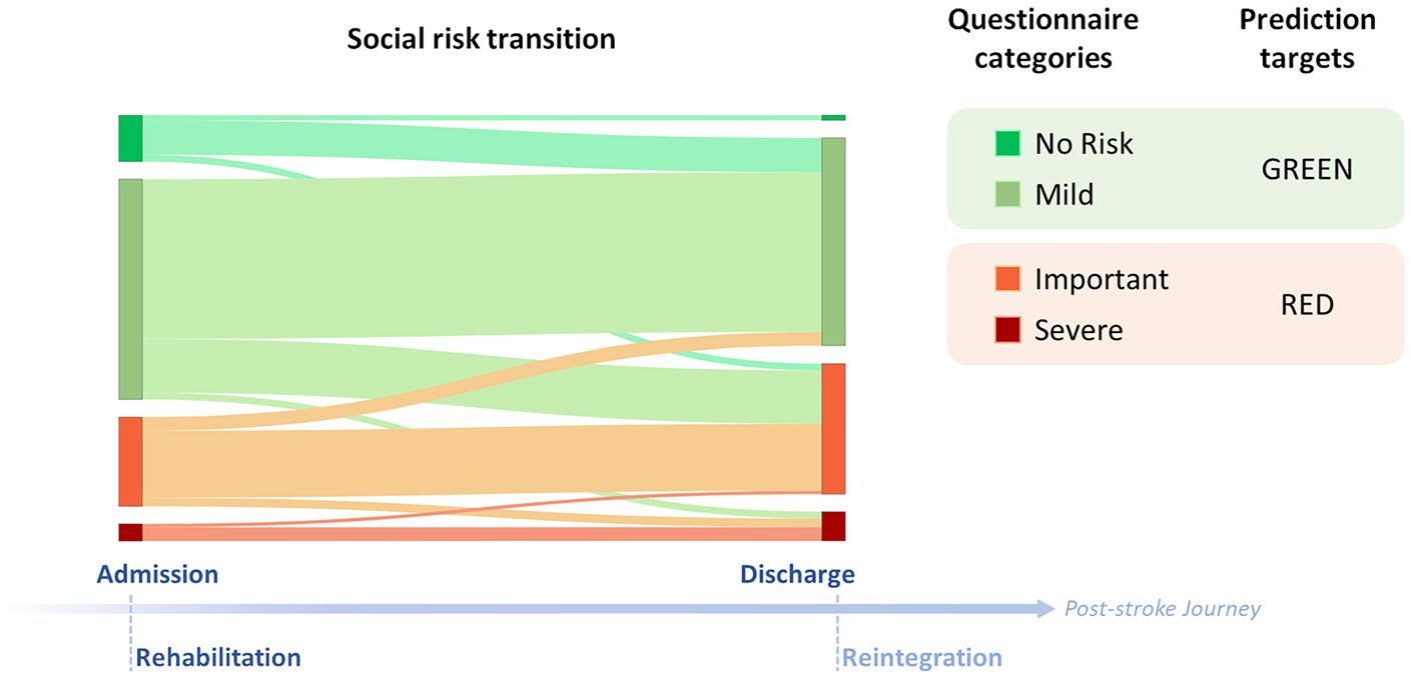
Clinical categories and distribution of patients from EVSF total scores for the training set.

On the basis of available demographic, diagnostic and questionnaire data at the admission of the patient to the Guttmann rehabilitation hospital, the patient cohort consisted of 217 patients and 16 variables for the modelling (Table 2). Although the Length Of Stay variable reported was the actual duration of the patients in rehabilitation from admission to discharge, this variable is estimated by clinicians at admission^20^. Length of stay varies greatly within Spain; for an older population with mean age of 79.6 ± 7.9 years, Pérez et al reports mean 61.6 ± 45.6 days for 9 facilities in Catalonia-Spain, however, younger patients, such as the patients in this study who are 30 years younger, are reported to stay longer^26,38, 39^. Hence, the longer length of stay (median 90 days) in this cohort is indicative of the poor functional status of this young, Spanish population (Table 2). The changes in social risk dimensions during patients stay at the hospital were previously examined in Cisek et al.; approximately a third of patients transitioned into another category by improving or worsening their social risk situation, and the majority of patients changed individual risk dimensions^35^. Since patients can undergo a social risk transition over the course of rehabilitation, the 16 admission variables were used to predict the level of social risk at discharge from rehabilitation in a binary classification, where patients in the no social risk and mild social risk categories were considered as having *negligible* social risk (GREEN), whereas patients in the important and severe social risk categories were considered as having *significant* social risk (RED) (Fig. 1). In the 217-patient cohort, there were twice as many male patients as female patients; there was no way to control for this sex ratio in the admitted patients or any gender bias in the referral from acute treatment units. There was a similar imbalance for the social risk classification (Table 2); nearly twice as many patients had negligible social risk (GREEN) than significant social risk (RED) at discharge from the hospital.

### Hold-out test set patient cohort

For validation purposes, demographic, diagnostic and data utilizing the EVSF questionnaire during the rehabilitation and reintegration of patients were recorded and collected at the Institut Guttmann (Barcelona, Spain) from 2020 through 2021 during a prospective study. The initial inclusion criteria for this cohort were the same as for the training set and consisted of adult patients 18–85 years of age at the time of stroke with an ischemic stroke diagnosis who were admitted within 3 weeks of the onset of symptoms, without any previous comorbidities leading to disability, and whose data was recorded within a week of admission and discharge. Similarly, exclusion criteria were any of the following: diagnosis of stroke in the context of another concomitant comorbidity (e.g., traumatic brain injury), a previous history of another disabling condition, patients with EVSF questionnaire performed more than 5 months post-injury, as well as more than 5 months stay at the rehabilitation hospital.

**Figure 2:**
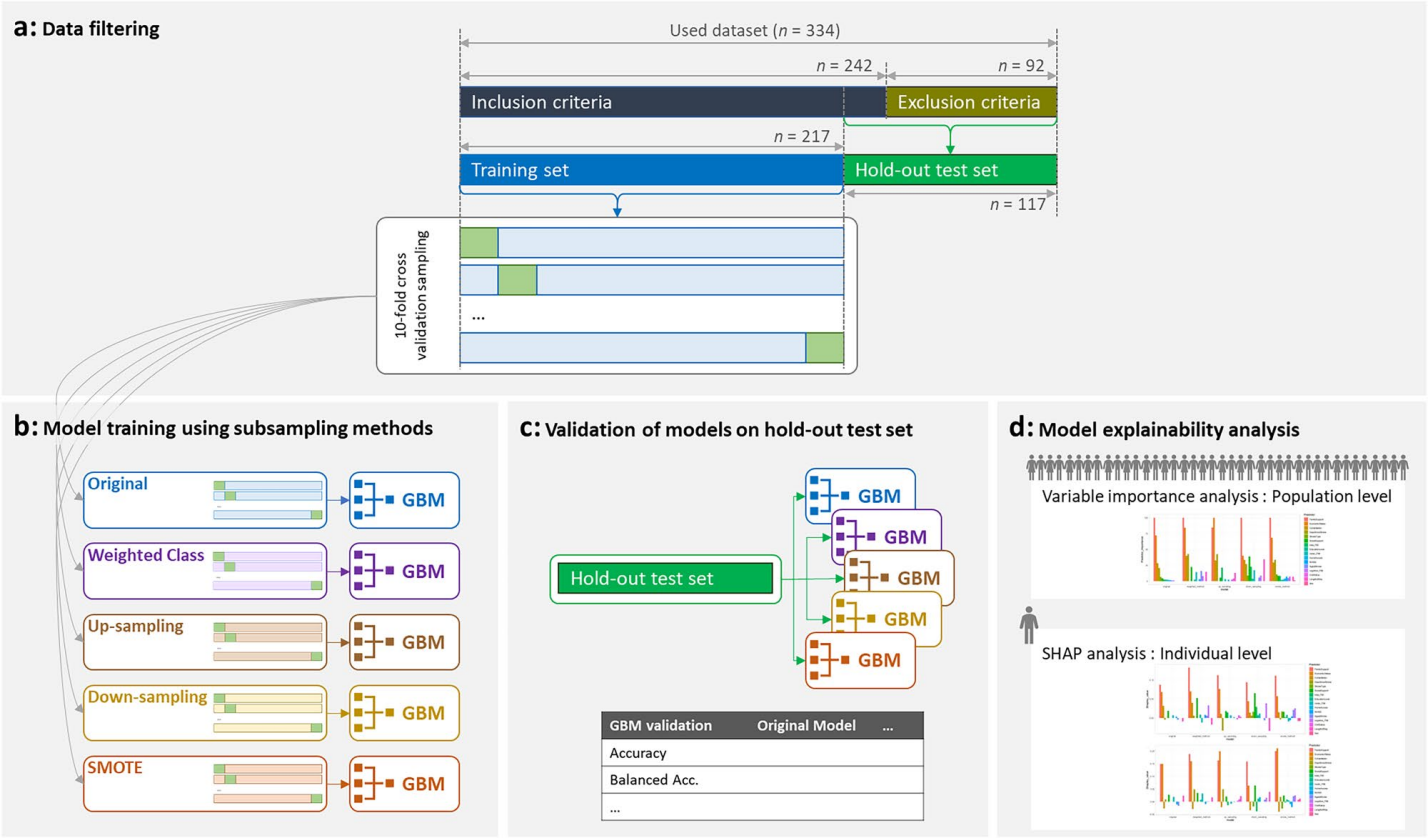
Predictive modeling framework. (**a**) Analysis begins with data filtering, using inclusion and exclusion criteria to partition data into the training set for 10-fold cross-validation sampling and hold-out test set for external validation. (**b**) Model training using GMB methodology tunes hyperparameters during cross-validation and selects the best models for each of the five subsampling methods. (**c**) Models are validated on the hold-out test set (data not used in model training) to evaluate performance and calculate metrics. (**d**) Model explainability analysis generates variable importance at the population level, as well as SHAP analysis to identify predictors of social risk at the individual level.

However, the difficulties caused by the Covid pandemic resulted in a reduced number of new patients being recruited for this prospective study for validation, only 25. Therefore, in addition to these 25 patients from the prospective study meeting inclusion criteria, an additional 92 patients, that were filtered out for model training due to exclusion criteria were added to the hold-out test set. The benefit of using patients with exclusion criteria for external validation is that it validates the utility and robustness of the models in a real-world clinical use case where patients at social risk may not meet inclusion criteria (specifically patients older than 85 years old, patients assessed more than 5 months post-stroke and patients with a longer length of stay at the rehabilitation hospital). Indeed, for these 92 patients, some had comorbidities and other disabling conditions in addition to the stroke diagnosis (data not shown), whereas Days Since Stroke and Length of Stay (Table 3) were significantly higher than other subjects, while other variables were similar to the rest of the cohort. Similarly, to the model training cohort, there is an imbalance in the hold-out test set dataset of the negligible (GREEN) and significant (RED) social risk patients, with twice as many GREEN than RED class patients. Table 3 shows the hold-out test set cohort information of the total 117 patients (25 from the prospective study with inclusion criteria plus 92 patients not used for training due to exclusion criteria). Data filtering according to inclusion and exclusion criteria is presented in Fig. 2a.

### Machine learning analysis

The ML analysis has two goals. The first is to create a prediction model that at the point of admission to rehabilitation can accurately forecast the level of social risk that an individual will experience at discharge. The second is to understand what are the factors that drive high social risk. We approach this second goal by analyzing what predictors are important in driving the models’ predictions for the entire cohort, as well as for an individual patient. The framework is presented in Fig. 2.

To create a binary classifier of significant (RED) or negligible (GREEN) social risk level at discharge, we used Generalized Boosted Regression Models (GBM)^40^ as implemented in R statistical software^41,42^. In a boosted ensemble methodology, a strong prediction model is built by combining a set of (potentially weaker) component models. The component models are built through successive iterations of model building over the training dataset^43,44^. At each iteration a new component model is trained, so as to pay particular attention to the errors the models already in the ensemble made on the training data, and is added to the ensemble. In contrast to other decision tree algorithms such as random forest, that generate ensembles of deeper independent trees, GBM generates sequential ensembles of shallow trees, improving performance incrementally (by reducing error in each iteration) instead of taking an average of all models^40^. Although shallow trees may be weak predictive models, they are “boosted” to produce a powerful ensemble, making GBMs efficient and powerful, especially for classification problems^45^.

Classification models can suffer from poor performance (poor model fit, or poor sensitivities and specificities) in the case that the target classes are imbalanced, such as the cohort in this study. We used four subsampling techniques to correct for this issue^46^. These four techniques were: incorporating weights of the classes into the cost function, (i.e., giving equal weight to both classes in binary prediction) without resampling the data; randomly up sampling (with replacement) the minority class to equal the size of the majority class; randomly down sampling and dropping the majority class samples so that it equals the size of the minority class, which results in model training on a subset of the total data; and hybrid sampling using the synthetic minority oversampling technique (smote) methodology which down-samples the majority class and synthesizes new data samples in the minority class by interpolating between existing minority class data samples. To robustly model the dataset and evaluate the importance of the variables as predictors, models were trained on the original dataset (not correcting for class imbalance), using a weighted cost function (giving equal weight to both classes), and with the up sampling, down sampling and smote method described above being applied to the data (Fig. 2b)^47,48^.

For each of these five experimental conditions we performed a 10-fold cross-validation process on the training data (further splitting the training data into 90% fold training set and 10% fold test set for each fold), where the subsampling techniques were applied inside each cross-validation fold on the 90% training subset of data, but not the 10% fold test set (Fig. 2a). Our motivation for using a cross-fold validation processes was first to find for each subsampling method the best hyperparameters of the GBM algorithm (discussed in more detail below), and second to create a baseline estimation of model performance that provides a comparator to contextualise the performance obtained on the hold-out test set. In terms of assessing model performance for each subsampling method, using a cross-validation methodology enables us to consider model performance across different training and validation sets and to report both a mean performance for each metric and a confidence interval across the 10 folds. To enable a more reliable estimation of model performance from cross-validation for each fold, the 10% test set, which was used to calculate performance metrics (confusion matrices and scores), was not adjusted using subsampling methods. For each subsampling method, once the hyperparameters for the GBM algorithm were fitted via cross-validation^49^, a final model was trained on the entire training dataset (with the subsampling method applied to the full dataset and using the corresponding fitted hyperparameters) and assessed on the independent hold-out test set.

All models were generated using the R statistical software^41,42^ using the GBM algorithm as implemented in the gbm package^46^ with a k-fold (k = 10) cross-validation with 10 repetitions with resampling method, and sampling (’up’, ’down’, ’smote’) and class weighing applied using the implementation from caret library^50^ (weights using the weights argument in the train function, and sampling methods using the sampling argument in the trainControl, ensuring that the subsampling step is correctly done inside of the cross-validation procedure as described in the paragraph above)^51^. Tuning hyperparameters ’shrinkage’ was held constant at a value of 0.1 and ’n.minobsinnode’ was held constant at a value of 10. The hyperparameter grid search explored via cross-validation included number of tress (n.trees = 50, 100, 150 and interaction depth = 1, 2, 3. The final hyperparameters used for the models were n.trees = 50, interaction.depth = 1 (except for smote where model interaction.depth = 2), shrinkage = 0.1 and n.minobsinnode = 10. The same random seeds were used for each model to ensure comparable results from the same cross-validation folds.

For each of the five experimental settings (original data, weighted cost function, up sampling, down sampling, and smote) a single “final” GBM model was trained using the best hyperparameters found for the data subsampling method via cross-validation by fitting the GBM model to all the training data. These final models were then validated by applying the independent 117 sample hold-out test set not used in the training in the predict() function to predict the social risk level for these samples (Fig. 2c). It is important to note that the subsampling techniques were only applied to the training portion of the data and the label distribution in the hold-out test set was not adjusted using these subsampling techniques. As a result, the model validation performance we report on the hold-out test set is indicative of model performance on a real data distribution. The validation on the hold-out test set gave a training-independent estimate of the real performance of the models, not only to compare the models but also to validate the models robustness on data samples outside of the training set inclusion criteria, thereby reflecting the variety of patients in real world scenarios.

For the cross-validation processes that were run for the resampling conditions (up sampling, down sampling, and smote) the resampling method was only applied to the training folds and not to the test set validation for each fold. Consequently, in all the cross-validation processes (irrespective of whether a resampling is applied to the training folds) each example in the training data is used exactly once as a test validation sample (i.e., it occurs in only one of the validation folds and only once in that validation fold). To construct the confusion matrices we recorded for each example in the training data whether the prediction returned for that example was a true-positive, false-positive, false-negative, of true-negative, and present the totals for each of these four outcome types across the 10-validation folds. We also report a range of performance metrics calculated across the 10-validation folds, including Accuracy, Recall, F1 score, Precision, and AUC (Recall and sensitivity are the same measure. We report both here as it is standard to report recall alongside precision and sensitivity alongside specificity)^52,53^. Some of the metrics, such as accuracy, focus on overall performance, others—such as F1, Sensitivity/Recall, Precision—prioritise performance on the minority/positive class (RED, significant social risk), and others—Specificity—prioritise performance on the majority/negative (GREEN, negligible social risk) class. Metrics Accuracy, Precision, Recall and F1 were calculated directly from the values reported in the confusion matrices, whereas metrics AUC, Sensitivity, Specificity and Balanced Accuracy were calculated by averaging over the metrics results obtained in each of the 10-validation folds.

### Prediction interpretation analysis

In addition to identifying the most important predictor variables for the models, it is also important to discern which variables contribute to the prediction of a particular class for an individual patient; i.e., it is crucial for clinicians to know which variables drive the social risk prediction outcome for that patient (Fig. 2d). To this effect, we applied a local interpretability analysis of a predictive model that was proposed by authors in the publication “A Unified Approach to Interpreting Model Predictions” called SHAP (SHapley Additive exPlanations)^54^, a model-agnostic approach based on Lloyd Shapley ideas for interpreting predictions. Unlike other expandability methodologies such as Local Interpretable Model-agnostic Explanations (LIME), SHAP is based on a strong theoretical basis, provides a full interpretation of a prediction, rather than an explainability prediction model. Moreover, it allows for contrastive explanations; instead of comparing a prediction to the average prediction of the entire dataset, it can be compared to a subset, such as a target class, or even to a single data point.

In brief, Shapley values are calculated on the prediction of a data point using the marginal contribution of a variable to a given model, i.e., a Shapley value is the average marginal contribution of that variable across all possible sets of variables (predictors)^54^. Therefore, variables with a high positive Shapley value are contributing more to the final prediction in contrast to negative values. In other words, Shapley values explain the distribution of the prediction results (classification) among the predictors. In the case that exact Shapley values are calculated for a single data point, they should add up to the difference between the prediction for that observation and the average predictions across the entire training set. Since SHAP calculates the average impact of adding a variable to the model by accounting for all possible subsets of the other variables, the computation time of exact Shapley values grows exponentially with the number of variables in the model. To improve computational efficiency, approximate Shapley value calculations consider the root-to-leaf paths in the trees that contain the target variable, and all the subsets within these paths. For this work we opted to use approximate Shapley values calculated using the Monte Carlo simulation approach described in^55^. The approximated Shapley values (nsim = 50) considering all the data points in the training dataset were calculated for each of the models using package fastshap^56^, indicating the contribution of each of the predictors to the negligible risk prediction (GREEN class) and the significant risk prediction (RED class).

## Results

**Figure 3:**
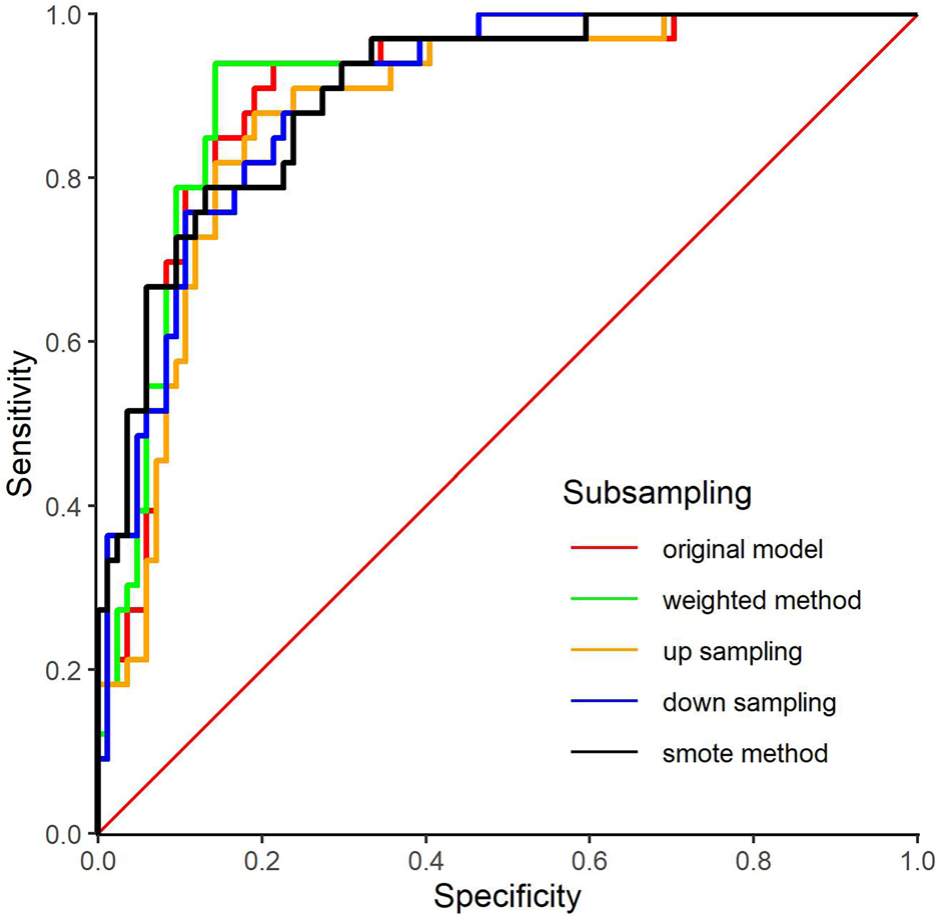
ROC AUC curves for each of the subsampling models.

Confusion matrices as well as standard classification model metrics including Accuracy, Recall, F1 score, Precision, were generated and the Area Under the Receiver Operator Characteristic Curve (ROC-AUC) as the evaluation metric for the best performing models (Tables 4 and 5). Corresponding ROC-AUC curves are presented in Fig. 3. Tables 4 and 5 present for each of the five experimental conditions the confusion matrices and a set of performance metrics calculated from the test sets across the validation folds during the 10-fold cross-validation process run on the training data.

**Table 4:** Confusion matrices for all models calculated across the 10-fold cross-validation process run on training data (top-left true-pos., top-right false-pos., bottom-left false neg., bottom-left true-neg.).

Training dataset label distribution				Original model	RED	GREEN
RED		GREEN		RED	37	13
62		155		GREEN	25	142
Weighted method		RED	GREEN	Up sampling	RED	GREEN
RED		53	26	RED	52	20
GREEN		9	129	GREEN	10	135
Down sampling		RED	GREEN	Smote method	RED	GREEN
RED		52	31	RED	52	16
GREEN		10	124	GREEN	10	139

**Table 5:** Model training performance statistics (bold font indicates the highest score for a metric).

GBM model statistics	Original model	Weighted method	Up sampling	Down sampling	Smote method
Accuracy	0.8249	0.8387	0.8618	0.8111	**0.8802**
Acc. 95% CI	(0.7677, 0.873)	(0.7829, 0.885)	(0.8086, 0.9047)	(0.7525, 0.8609)	(0.8294, 0.9202)
AUC	0.841	0.842	0.831	0.827	**0.843**
Sensitivity	0.5968	**0.8548**	0.8387	0.8387	0.8387
Specificity	**0.9161**	0.8323	0.871	0.8	0.8968
Precision	0.74	0.6709	0.7222	0.6265	**0.7647**
Recall	0.5968	**0.8548**	0.8387	0.8387	0.8387
F1	0.6607	0.7518	0.7761	0.7172	**0.8**
Balanced accuracy	0.7565	0.8435	0.8548	0.8194	**0.8677**

### Model performance

Looking at the performance metrics in Table 5 all the models have good mean performance across the validation sets in the cross-fold validation, accuracy in the range of 0.811–0.880, and AUC in the range 0.827–0.843, with the smote sampling model outperforming all other models followed by the up sampling model. For each model we calculated the 95% confidence interval around its mean accuracy using the accuracies obtained by the model across the folds as the population. These confidence intervals overlap (i.e., the lower end of the CI range for the best model is lower than the high end of the CI range for the weakest model) which suggests that at the 95% confidence level there is no statistical difference between the accuracies of the models. However, if we consider measures such as F1, balanced accuracy, sensitivity, specificity, we do see differences between the models. This is because these measures explicitly weigh for class distribution and/or performance on the minority class (in this instance significant social risk).

What is of importance to clinicians is to identify patients with significant social risk (RED class). Sensitivity (also known as recall) is the critical performance metric here because it measures out of all the patients with significant social risk (positive class) how many of these did the model predict as having significant social risk. On this metric, the original model performs much worse than the other models, and the weighted method model has the best performance. In fact, the original model has the lowest sensitivity and the highest specificity of all models, suggesting that the original model is over predicting the majority class. This difference in model performance on the significant social risk class is also evident in the confusion matrices in Table 4. The model trained on the original data, with no adjustment for class imbalance, either in terms of cost function class weighting or resampling, only correctly identified 37 out of the 62 individuals who had significant social risk, by comparison the other models correctly identified 53 or 52 of these cases. From among these other four approaches, the smote method has comparable recall/sensitivity with the others and has better precision (very few false positives) resulting in the best overall F1. The smote method also results in the best overall balanced accuracy.

**Table 6:** Confusion matrix for hold-out test set validation (top-left true-pos., top-right false-pos., bottom-left false neg., bottom-left true-neg.).

Hold-out test set label distribution				Original model	RED	GREEN
RED		GREEN		GREEN	26	9
33		84		RED	7	75
Weighted method		RED	GREEN	Up sampling	RED	GREEN
GREEN		31	24	GREEN	30	23
RED		2	60	RED	3	61
Down sampling		RED	GREEN	Smote method	RED	GREEN
GREEN		31	30	GREEN	26	18
RED		2	54	RED	7	66

**Table 7:** Model validation performance metrics for hold-out test set (bold font indicates the highest score for a metric).

GBM validation	Original model	Weighted method	Up sampling	Down sampling	Smote method
Accuracy	**0.8632**	0.7778	0.7778	0.7265	0.7863
95% CI	(0.7874, 0.9198)	(0.6916, 0.8494)	(0.6916, 0.8494)	(0.6364, 0.8048)	(0.7009, 0.8567)
AUC	0.891	**0.909**	0.881	0.899	0.904
Sensitivity	0.7879	**0.9394**	0.9091	**0.9394**	0.7879
Specificity	**0.8929**	0.7143	0.7262	0.6429	0.7857
Precision	**0.7429**	0.5636	0.566	0.5082	0.5909
Recall	0.7879	**0.9394**	0.9091	**0.9394**	0.7879
F1	**0.7647**	0.7045	0.6977	0.6596	0.6753
Balanced Accuracy	**0.8404**	0.8268	0.8176	0.7911	0.7868

### Model validation

Confusion matrices as well as standard classification metrics including AUC, Accuracy, Sensitivity and Specificity, were generated for the validation on the independent hold-out 117-patient dataset and are presented in Tables 6 and 7.

On the hold-out test set the model trained using the original data distribution obtains the highest overall accuracy, 0.8632. However, the overlap of the confidence intervals of all the models indicates that none of the accuracy scores are significantly different at the 95% confidence level. Comparing these accuracy scores with the ones obtained through the cross-validation process the accuracy of the original model on the test set is higher than the accuracy obtained in the cross-validation data (although this difference is not statistically significant at the 95% level). By comparison, the accuracies of the other models all drop between the cross-validation and hold-out test set. These drops are in the range of 0.06–0.09 (again none of these drops are statistically significant at the 95% level).

Regarding AUC all the models have similar performance, in the range of 0.881–0.909. Interestingly all the models obtained a higher AUC score on the test set than the models trained in the corresponding setting during cross-validation, these increase range between 0.05 and 0.07. Although hold-out test set AUC values were generally slightly higher than those obtained during cross-validation, the test set and cross-validation accuracies are very comparable indicating that all models had a similarly stable and robust performance in both settings. As in the cross-validation setting the identification of individuals with significant social risk is of primary performance, and consequently, the performance of the models in terms of sensitivity/recall is of particular concern. On the hold-out test set, sensitivity was higher than specificity for all models (except the model trained on the original data) suggesting that applying a class weighting or resampling method does produce models that are more sensitive to the significant social risk class. The model trained using the weighted cost function method obtains the joint highest score for sensitivity on the test set (0.9394), and the highest score for sensitivity in the cross-validation setting (0.8548), and the highest AUC on the test set (0.909). However, the precision of these weighted models is low. Indeed, the model trained on the original data distribution has the highest accuracy, specificity, precision, F1, and balanced accuracy on the test set. This suggests that there is a trade-off between sensitivity and specificity and our results do not indicate a clear winning method on this task.

Overall, our results do indicate that it is possible to train a model that can accurately predict social risk at discharge based on the information available at admission to rehabilitation. The mean accuracy of all the models based on cross-validation and the test set results is 0.8148 (min 0.7265, max 0.8802), the mean F1 is 0.7208 (min 0.6596, max 0.8), the mean sensitivity is 0.8331 (min 0.5968, max 0.9394), and the mean specificity is 0.8078 (min 0.6429, max 0.9161). Given this, in the following sections, we analyze these models to better understand what variables they use in making the predictions, so as to gain insight into the drivers of social risk.

### Understanding the drivers and predictors of social risk

Beyond knowing which individuals are at social risk it is also useful to know what the general drivers of social risk are and what are the factors that are driving social risk in a particular individual. One way of understanding the relative importance of variables in terms of their contribution to social risk is to analyse the importance of the variables to a model’s output. This analysis can be done at two levels (Fig.2d). First, we can analyse the importance of a variable to a prediction model’s performance across the entire dataset. This type of analysis can inform our understanding of what variables are most important in general to social risk at the population level. The second analysis we can do is to analyse what variables are most important in determining the social risk for a particular individual. This analysis can support the design of a personalised programme of interventions to reduce the social risk for that individual. In this section, we present both of these types of analyses using the models developed and validated in the above sections. First, we present general variable importance, where predictors are ranked for each model, followed by model explainability, where we explain how particular variables drive social risk for an individual.

#### Variable importance

**Figure 4:**
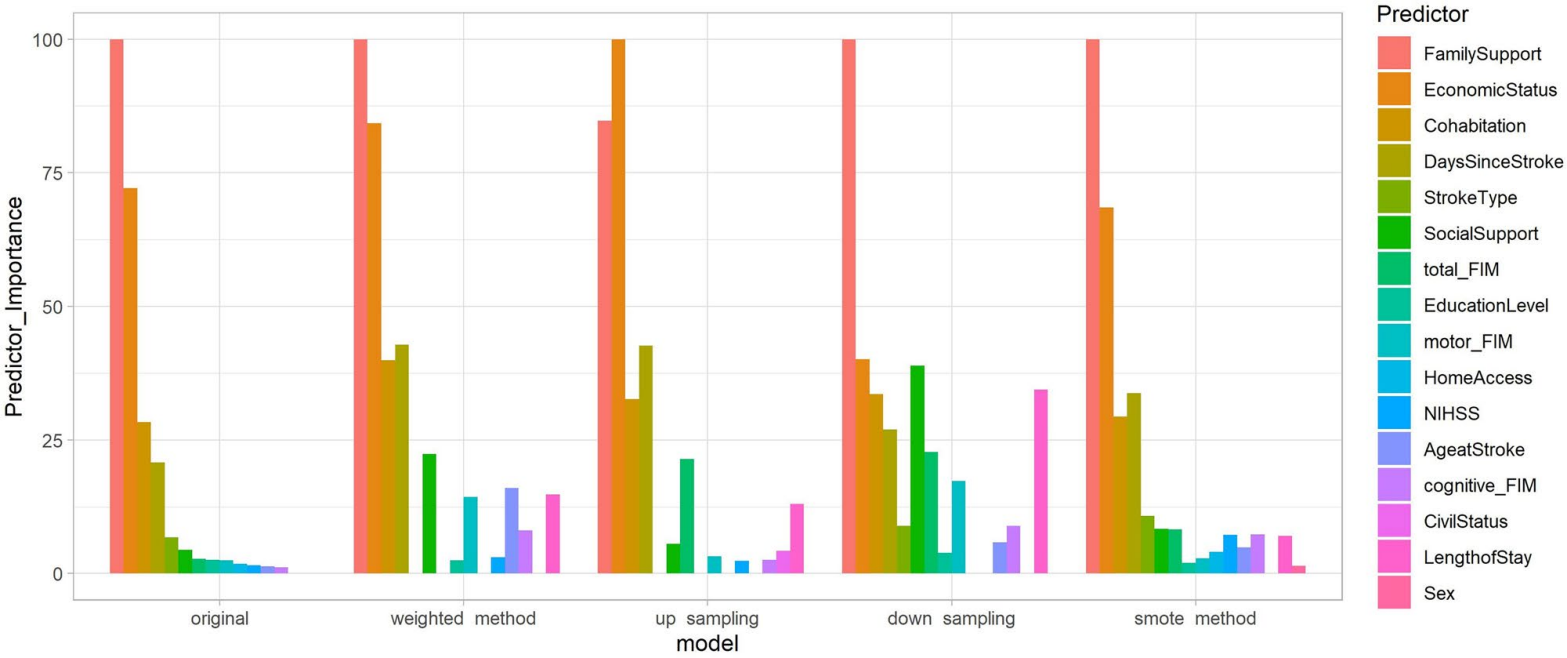
Rankings of predictor variable importance for the entire training set cohort.

In addition to accurately identifying which patients are at social risk, it is also useful to understand what the primary factors are that drive social risk, as this informs the design of appropriate interventions to reduce the social risk. For this analysis for each of the 5 models that were run on the hold-out test set we calculated the relative influence of each predictor variable, i.e., whether that variable was selected to split on during the tree-building process, and how much the squared error (over all trees) improved (decreased) as a result. The intuition being that the more a variable contributes to the outputs of an accurate model the more important the variable is in terms of the phenomenon the model predicts. This variable importance analysis also acts as a sense check of model performance (i.e., if the model relies on variables that domain knowledge would indicate are not important this would suggest that the model may be overfitting to the specifics of the data sample used to create the model). Figure 4 presents the results of the variable importance analysis for each of our 5 models. This analysis revealed that although variable importance of all of the 16 predictors varied for each of the models, nevertheless, four predictors consistently retained their high importance value and rank order: Family Support and Economic Status, as well as with lesser importance but same ranking, Cohabitation and Days Since Stroke. Interestingly, the Sex variable did not have any notable importance for most of the models, which may reflect the imbalanced ratio of men to women.

#### Model explainability

**Figure 5:**
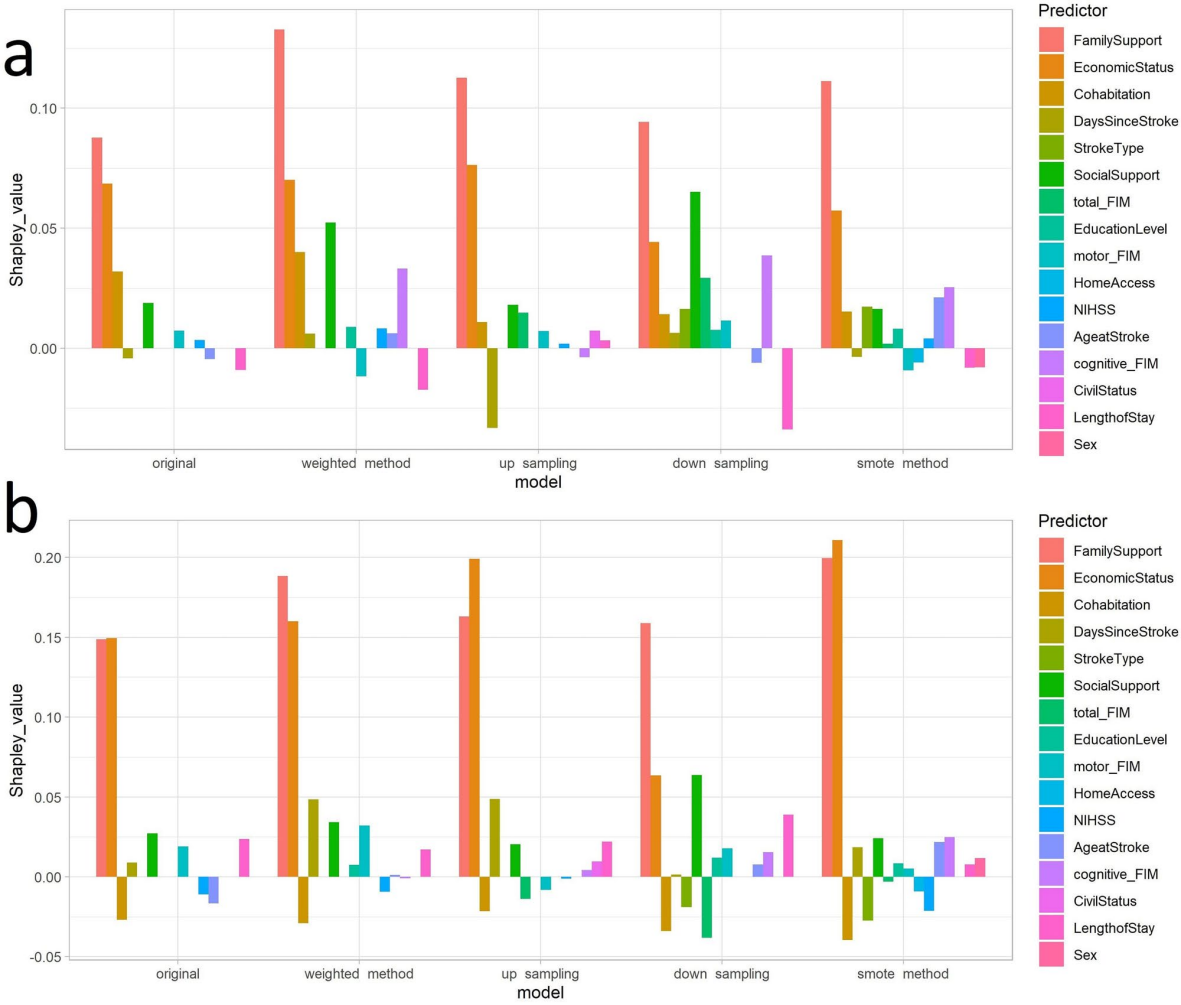
Approximate Shapley values (**a**) GREEN (**b**) RED for a single individual with strong negligible risk prediction.

**Figure 6:**
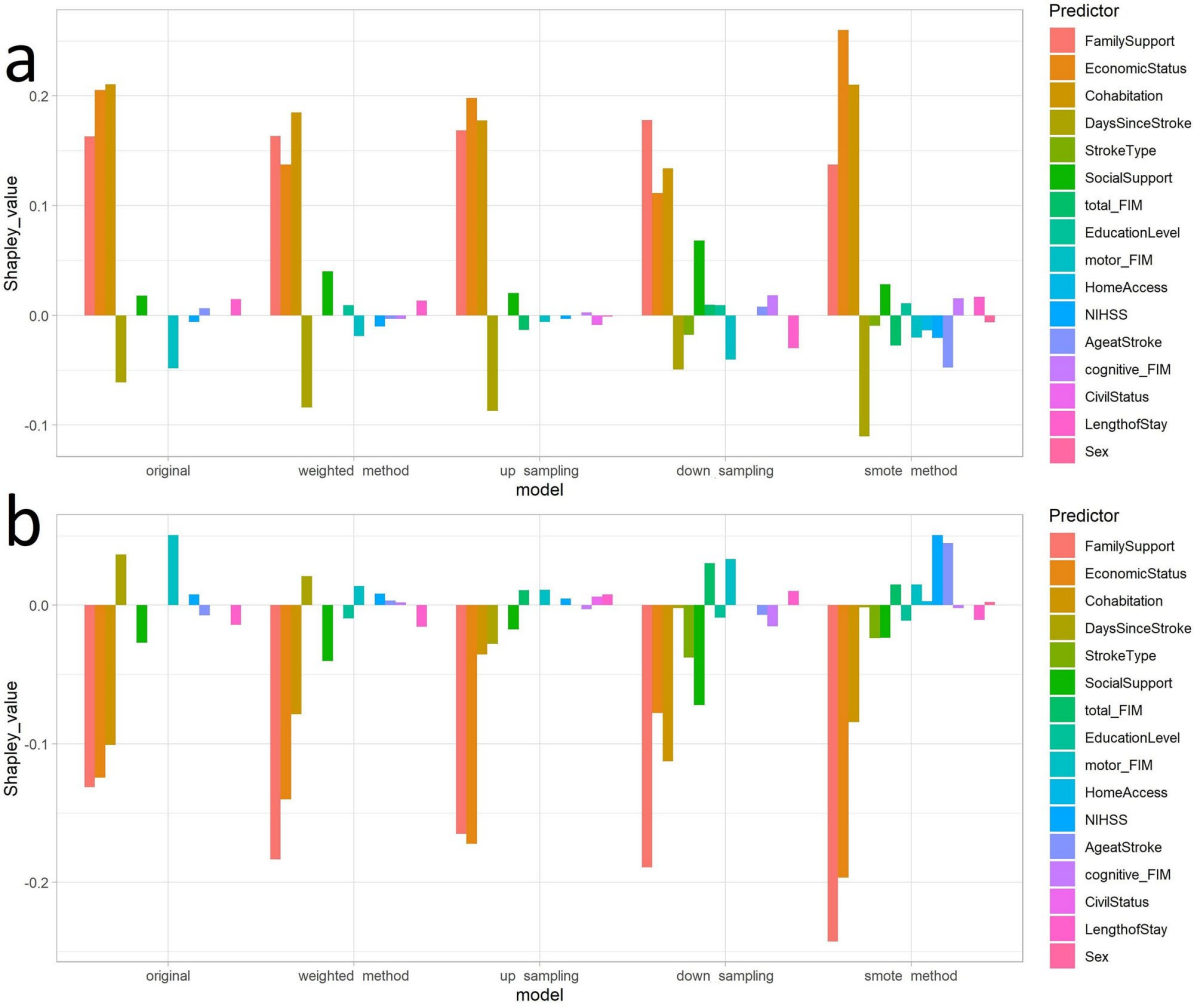
Approximate Shapley values (**a**) GREEN (**b**) RED for a single individual with weak severe risk prediction.

In binary classification tasks, predictions may fall near the classification threshold, therefore, the idea is that by using SHAP for establishing the contribution of each variable to either the GREEN or the RED classes separately, rather than as one population, we may elucidate the drivers of social risk for predictions near the threshold (50%) more clearly. In other words, for a given individual we can calculate the Shapley values for the variables under the assumption that the prediction of the model would be GREEN classification, and then calculate a separate set of Shapley values for the variables under the assumption that the prediction of the model would be a RED classification. In Figs. 5 and 6, we present the variables contributing to social risk for the 5 models of two random individuals from the training dataset. Figure 5 presents the results of a patient predicted with a probability of ${\sim }$ 90% across all models to have negligible risk (GREEN) whereas Fig. 6, presents the results of a patient predicted with a probability of ${\sim }$ 60% across all models to have significant risk (RED). For the first patient predicted to have negligible social risk with very strong probability, both the approximate SHAP results in the GREEN and RED plots mainly had positive predictor contributions to the prediction, meaning that these variables contribute to negligible social risk, and should these same variables change, they would contribute to significant social risk prediction. In the original model the variables Family Support and Economic Status made the largest contributions to the model’s predictions in both the GREEN and RED classification scenarios. This was also true for all other models (except the down-sampling model). In the down-sampling model, Social Support was the second highest contributing variable after Family Support. Except for the smote model, Sex and other demographic variables, which were similar to the variable importance rankings, were not notably predictive of social risk for this patient. Overall, this suggests that Family Support and Economic Status were the main drivers of the (negligible) social risk for this patient, and that Social Support also contributed to this outcome for this patient.

For the second patient predicted to have significant social risk with albeit close to the classification threshold at ${\sim }$ 60%, both the approximate SHAP result in the GREEN and RED plots mainly had opposite predictor trends (i.e., the positive Shapley values drove the negligible risk prediction Fig. 6a, whereas the negative Shapley values did not contribute to the significant social risk prediction (Fig. 6b). For all the models, Family Support, Economic Status and Cohabitation had the greatest contributions to negligible risk (Fig. 6a, and the predictor Days Since Stroke also had an important contribution. However, the prediction for this patient was that of significant social risk (RED class), where depending on the model, mainly clinical variables contributed to this final prediction (Fig. 6b: for original and weighted method models the greatest predictors were Days Since Stroke and motor FIM and for up sampling and down sampling models the predictors were total FIM and motor FIM and for the smote method model the predictors were NIHSS and Age @ Stroke. Similarly to the other patient SHAP result (Fig. 5), except for the smote method model, demographic variables such as Sex had a negligible contribution to the class prediction.

## Discussion

Successful rehabilitation includes identifying patients at risk of poor reintegration trajectories and minimizing patient fragility by reducing social risk^4,6, 14^. From the clinical perspective, this entails adequately supporting patients in the areas that contribute to the individual’s overall social risk^11–13^. A few observational studies have explored socioeconomic, environmental and demographic factors predictive of the discharge destination after rehabilitation (i.e., home, hospital, care home), and found that several factors, such as cohabitation with a caregiver, family support, and marital status, were influential in discharge planning and destination^16–19, 57–62^. Although these studies identify risk factors for patient populations, they do not forecast the level of social risk nor the specific variables contributing to that risk for an individual patient, despite a great need for such a predictive tool^63^. To the best of our knowledge, there are no publications predicting the level of an individual patient’s social risk based on an ad hoc questionnaire routinely integrated into the clinical practice for assessing social factors (EVSF).

To this effect, this study applied a GBM ML methodology to a 217-patient cohort of mostly young, male, ischemic stroke survivors who were evaluated for their functional independence (FIM assessment) as well as social risk (EVSF questionnaire), to forecast social risk upon discharge from the rehabilitation hospital. Due to the target class imbalance (approximately twice as many negligible social risk patients (GREEN) than significant risk patients (RED)), several binary classifiers were built, including an original model (not correcting for class imbalance), as well as a weighted model and three other models utilizing subsampling methodologies to balance classes. The performance of the models was accurate as well as very comparable on the basis of AUC, even for the original model, however, because the prediction of patients with significant risk is essential to clinicians, model sensitivity was one of the most crucial metrics (Table 5). Therefore, depending on the characteristics of the cohort used as the training data for social risk classification, the performance of the resulting models with and without subsampling techniques may vary and should be validated on independent hold-out test sets to assess their performance.

The population level variable importance from the predictive models, as well as individual predictor contribution to GREEN and RED class prediction using SHAP methodology, both mainly indicated Family Support and Economic Status, rather than demographic variables such as Sex, Educational Level or Civil Status, contributed to social risk prediction. Interestingly, our previous work utilizing visualizations to explore this data^35^ found that Family Support was an important dimension for identifying patient risk at discharge, however, Economic Status did not emerge as an important variable; this predictor was only detected using ML (suggesting that the importance of this variable arises from its interaction with other variables). However, other predictor variables varied in rank and contribution to the predictions, which is likely due to the complex and multifactorial nature of social risk, where a combination, rather than a single risk factor may be increasing social risk for individuals. Moreover, in order to discern which variables contributed to social risk for a particular individual rather than the whole cohort, we utilized explainability metrics using approximate Shapley values in order to assign contribution values of each predictor variable to the overall prediction for two random patients: first strongly predicted to have negligible risk (${\sim }$ 90% probability GREEN) and the second predicted to have significant risk (${\sim }$ 60% probability RED). Interestingly, for the first patient (Fig. 5), adequate Family Support and Economic Status contribute to that patient’s high probability of negligible social risk and should these factors change, they would mainly contribute to the patient’s increased social risk (Fig. 5b), rather than demographic or clinical factors, such as the patient’s age or functional status. In contrast, for the second patient (Fig. 6), whose prediction was closer to the classification threshold, the significant social risk prediction was driven by the patient’s clinical outcomes, mainly functional status (motor FIM and total FIM), rather than sociofamiliar factors (Fig. 6b). The personalized intervention for this patient may include a longer length of stay at the hospital to improve clinical outcomes or a professional carer at home. This further supports the use of a predictive tool for personalized forecasting an individual patient’s social risk.

### Strengths and limitations

Individualized intervention strategies for patients at social risk are necessary for successful rehabilitation for many individuals and planning an optimal length of rehabilitation^64,65^. Indeed, optimizing the length of stay to the needs of the individual patient not only offers opportunity for minimizing social risk after discharge, but also the right amount of time for implementing a personalized intervention during rehabilitation^20,38^. In the clinical setting, key challenges to developing individualized interventions include first identifying individual patients at social risk and identifying the specific factors that contribute to this risk (variable predictors)^35^. The machine learning analysis in this work overcomes these two challenges by stratifying patients by their social risk, rank ordering the factors of social risk by the general importance to the prediction of risk, and finally, for specific individuals at risk we identify the most important predictors of risk for each of these individuals. This third contribution of identifying the specific factors of risk for an individual is, we believe, a particularly noteworthy novelty of this work. The practical application of the results can in turn optimize the decision-making process during reintegration, by specifically tailoring intervention strategies to the patient, to minimize their social risk, improve their outcomes and quality of life during reintegration. Another strength of the study design focusing on the period between admission and discharge from the rehabilitation hospital is minimization of confounder effects. Post-discharge, stroke survivors and carers are faced with immense emotional, health and social related challenges, such as fragility (patient falling into depression and retreating from social life) or a recurrent stroke and these additional factors can contribute to the improvement or deterioration of social risk dimensions^66,67^. Furthermore, the GBM and SHAP methodologies implemented in this work are flexible, accurate and reliable and for small to medium datasets have relatively low complexities, enabling deployment with minimal resources and computational time. It is feasible that this analysis can be implemented as an app for clinicians.

The main limitation of this study stems from the size of the dataset^68–70^. In rehabilitation hospitals, there is no way to control for either the number of stroke patients admitted in a given time period or the gender ratio (this cohort has twice as many male as female patients) because patients are referred from acute treatment units. Moreover, the Covid pandemic resulted in a reduced number of new subjects for the hold-out test set validation. However, identifying adequate sample sizes for predictive modeling is not a trivial task, because large training datasets do not automatically guarantee strong predictive models. The consensus between various guidelines for developing robust predictive models is applying a hold-out test set to validate models. Hold-out validation is regarded to be one of the most definitive and reliable strategies of evaluating the accuracy and overall performance of prediction models^47,71, 72^. It is a robust safeguard against overfitting, because it tests the models against new (unseen) data samples to give a realistic model performance evaluation^73^, such as the hold-out test set validation in this study. Nevertheless, additional external validations on different cohorts (subjects with hemorrhagic stroke, comorbidities, from other countries, etc.) would evaluate the generalizability and robustness of these models to other populations and geographical regions.

## Conclusions

There is a lack of studies, especially including social aspects in young patients after stroke, despite the growing incidence of stroke in young populations^26,65, 74^. In this study, for the training cohort of patients of the Catalonia region of Spain, consisting of mostly male, young ischemic stroke patients, despite the prevalence of individuals in negligible social risk class upon discharge from the hospital, ML modeling of this data revealed that predictors contributing to significant social risk were primarily Family Support and Economic Status, as well as Cohabitation and Days Since Stroke, with lesser contribution of other predictors, such as the FIM and specifically no notable contribution from the Sex of the patient. Model validation on an additional patient dataset that was not part of model training reflects the actual usage of the models by clinicians for patients that may not meet inclusion criteria and confirmed the utility of the models in the real-world clinical scenario. However, due to the target class imbalance, as well as an imbalance in the numbers of men and women in the cohort, leaves room for future studies with a larger and more balanced cohort. This highlights that social risk is a complex and multifactorial phenomenon that can vary significantly for an individual over the course of stroke rehabilitation and reintegration and so it is important to understand what can drive these variations and also to be able to predict this variation so that appropriate support measures can be put in place in a timely manner.

## Data Availability

The models developed and/or analysed during the current study are available in the Gitlab repository, https://gitlab.com/precise4q-tud.

## Abbreviations

ADL: Activities of daily living
EVSF: Escala de Valoracion Socio Familiar
GBM: Generalized boosted regression models
FIM: Functional independence measure
ICF: International classification of functioning, disability, and health
LIME: Local interpretable model-agnostic explanations
ML: Machine learning
NIHSS: National institutes of health stroke scale
ROC-AUC: Area under the receiver operator characteristic curve
SHAP: Shapley additive explanations
